# Both the Positioned Supplemental or Night-Interruptional Blue Light and the Age of Leaves (or Tissues) Are Important for Flowering and Vegetative Growth in Chrysanthemum

**DOI:** 10.3390/plants13202874

**Published:** 2024-10-14

**Authors:** Jingli Yang, Jinnan Song, Yoo Gyeong Park, Byoung Ryong Jeong

**Affiliations:** 1 Weifang Key Laboratory for Stress Resistance and High Yield Regulation of Horticultural Crops, Shandong Provincial University Laboratory for Protected Horticulture, College of Jia Sixie Agriculture, Weifang University of Science and Technology, Shouguang 262700, China or yangmiaomiaode@gmail.com (J.Y.); jinnansong93@gmail.com (J.S.); 2 Department of Horticulture, Division of Applied Life Science (BK21 Four), Graduate School, Gyeongsang National University, Jinju 52828, Republic of Korea; ygpark20@korea.kr; 3 National Institute of Biological Resources (NIBR), 1008-11, Sangnam-ro, Sangnam-myeon, Miryang-si 50452, Republic of Korea; 4 Division of Horticultural Science, College of Agriculture and Life Sciences, Gyeongsang National University, Jinju 52828, Republic of Korea

**Keywords:** *Chrysanthemum morifolium*, floral signal, leaf age, photoperiodism, positioning light, shoot tip, supplementary or night-interruption blue light, vegetative growth

## Abstract

In this study, the effects of supplemental or night interruptional blue light (S-BL or NI-BL) positioning on morphological growth, photoperiodic flowering, and expression of floral genes in *Chrysanthemum morifolium* were investigated. Blue light-emitting diodes (LEDs) at an intensity of 30 μmol·m^−2^·s^−1^ photosynthetic photon flux density (PPFD) were used for 4 h either (1) to supplement the white LEDs at the end of the 10 h short-day (SD10 + S-BL4) and 13 h long-day conditions (LD13 + S-BL4), or (2) to provide night interruption in the SD10 (SD10 + NI-BL4) and LD13 (LD13 + NI-BL4). The S-BL4 or NI-BL4 was positioned to illuminate either the shoot tip, the youngest leaf (vigorously growing the third leaf from the shoot tip), or the old leaf (the third leaf from the stem base). In the text, they will be denoted as follows: SD10 + S-BL4-S, -Y, or -O; SD10 + NI-BL4-S, -Y, or -O; LD13 + S-BL4-S, -Y, or -O; LD13 + NI-BL4-S, -Y, or -O. Normally, the LD13 conditions enhanced more vegetative growth than the SD10 periods. The growth of leaves, stems, and branches strongly responded to the S-BL4 or NI-BL4 when it was targeted onto the shoot tip, followed by the youngest leaf. The SD10 + S-BL4 or +NI-BL4 on the old leaf obviously suppressed plant extension growth, resulting in the smallest plant height. Under LD13 conditions, the flowering-related traits were significantly affected when the S-BL4 or NI-BL4 was shed onto the youngest leaf. However, these differences do not exist in the SD10 environments. At the harvest stage, other than the non-flowered LD13 treatment, the LD13 + S-BL4 irradiating the youngest leaf induced the most flowers, followed by the shoot tip and old leaf. Moreover, LD13 + NI-BL4 resulted in the latest flowering, especially when applied to the shoot tip and old leaf. However, the SD10 + S-BL4 or + NI-BL4 irradiated the shoot tip, youngest leaf, or old leaf all significantly earlier and increased flowering compared to the SD10 treatment. Overall: (1) Generally, vegetative growth was more sensitive to photoperiod rather than lighting position, while, during the same photoperiod, the promotion of growth was stronger when the light position of S-BL4 or NI-BL4 was applied to the shoot tip or the youngest leaf. (2) The photoperiodic flowering of these short-day plants (SDPs) comprehensively responded to the photoperiod combined with blue light positioning. Peculiarly, when they were exposed to the LD13 flowering-inhibited environments, the S-BL4 or NI-BL4 shed onto the leaves, especially the youngest leaves, significantly affecting flowering.

## 1. Introduction

The transition from the vegetative to the reproductive stage, known as flowering, is considered to be a pivotal developmental stage in the life cycle of plants. This process is governed by internal gene networks and external environmental factors such as photoperiod [1]. Changes in photoperiod are one of the most significant and reliable signals for plants to reproduce in favorable seasons. The specific photoperiod is crucial for obligate-type plants to initiate responses [2]. *Chrysanthemum morifolium* is a type of obligate short-day plant (SDP), and its flowering occurs only when the duration of night exceeds a critical minimum (about 12 h) [3]. The specific differences in the photoperiodic sensitivity of flower formation depend on the species or culture.

Light is widely recognized as a key regulator of flowering in numerous plant species. Plants perceive the quality of light through photoreceptors, which fall into categories such as phytochromes, cryptochromes, phototropins, members of the Zeitlupe (ZTL/FKF1/LKP2) family, and the UV-B-absorbing UVR8 [4,5]. Collectively, these photoreceptors regulate photomorphogenesis across a wide range of wavelengths. Phytochromes respond to red and far-red light, while cryptochromes and phototropins absorb blue and UV-A lights [4]. Cryptochromes impact early growth, establishment, stem elongation, and the regulation of seedling photoperiodic responses [6,7]. Phototropins (two in *Arabidopsis thaliana* L.) regulate phototropism, chloroplast light-avoidance, and accumulation movements, hypocotyl growth inhibition, stomatal aperture, and leaf expansion [6,7].

How does a plant transmit signals to induce flowering after sensing the light? A transmissible factor known as florigen is synthesized in the leaves, serving as the vector of received photoperiodic signals. The concept of “florigen” for improving flowering was first proposed by Chailakhyan in 1936, based on a chrysanthemum experiment [8]. Currently, numerous studies have documented the synthesis of FT and its orthologs in the leaves of several species, where they function as florigens [9,10,11,12]. In *Arabidopsis*, FT moves into the shoot apical meristem (SAM) through the phloem and forms a transcriptional complex with FD, a crucial bZIP transcription factor. This complex then activates the floral regulator genes *FRUITFULL* (*FUL*) and *APETALA 1* (*AP1*), leading to flowering [13,14]. *FT* encodes a small protein similar to phosphatidylethanolamine-binding protein (PEBP-like) that acts as a florigen, regulating both activation and repression of flowering in the PEBP family. There are five additional members of the *FT* gene family in *Arabidopsis*: *TERMINAL FLOWER 1* (*TFL1*), *MOTHER OF FT AND TFL1* (*MFT*), *BROTHER OF FT AND TFL1* (*BFT*), *TWIN SISTER OF FT* (*TSF*), and *Arabidopsis thaliana CENTRORADIALIS* homolog (*ATC*). Several studies on the FT family have shown that FT and TSF function as floral activators [15,16,17], while TFL1, ATC, and BFT act as floral repressors [18,19,20]. MFT is also associated with seed germination [21]. Additionally, ATC and TFL1 are expressed in vasculature tissue and shoot apex, despite being non-cell-autonomous [22,23]. Floral repressors, also known as antiflorigenic stimuli, are synthesized in leaves [24]. This has been confirmed by classical physiological experiments showing flowering inhibition in tobacco cultivars with grafted leaves under non-floral-inductive light-duration environments [25]. Anti-floral factors have also been observed in chrysanthemum leaves under unfavorable day-length conditions for flowering [26].

Technical skills are commonly used in chrysanthemum cultivation to manipulate flowering time through methods such as blackouts, artificial lighting, day-length extension, and night interruption (NI) to ensure consistent production of marketable flowers. Light supplementation can be achieved by adding valuable light to the regular light source or extending the day length with extra light [27]. NI interrupts darkness with lighting, creating modulated long-day (LD) environments [28,29]. Higuchi et al. found that under short-day (SD) conditions with white light, NI treatment with monochromatic red light effectively inhibits flowering in chrysanthemums, while monochromatic blue or far-red light is less effective [30]. Additionally, supplying 4 h of low-level blue light either as supplementary or NI in SD conditions resulted in no significant differences compared to a normal SD environment [31]. In LD conditions, non-flowered plants flowered after being treated with low-intensity S-BL4 or NI-BL4, delaying flower bud formation [31,32]. However, extending natural sunlight during the first 11 h of the photoperiod with either red or blue sole light inhibited flowering in *Chrysanthemum morifolium* [33]. Therefore, cultivar-specific or other subtle details such as intensity, photoperiod, supplementary, or NI can affect the flowering response to blue light.

Previous studies have shown that the quality and positioning of night-interruptional light (NIL) significantly affect flowering and extension growth in chrysanthemums [34]. Short periods of S-BL or NI-BL with various intensities effectively regulate the photoperiodic flowering of this qualitative SDP [35,36]. No studies have yet been reported on the effects of lighting position of specific intensified-S-BL or -NI-BL and the age of leaves (or tissues) exposed to the photoperiodic light treatments in vegetative growth and flowering in chrysanthemums. This investigation contributes to the research model of photoperiodic flowering and growth regulation in response to different light treatments in obligate SDP *Chrysanthemum morifolium*. We hope our findings can provide potential applications for floricultural crop production.

## 2. Results

### 2.1. Growth and Flowering

**Figure 1:**
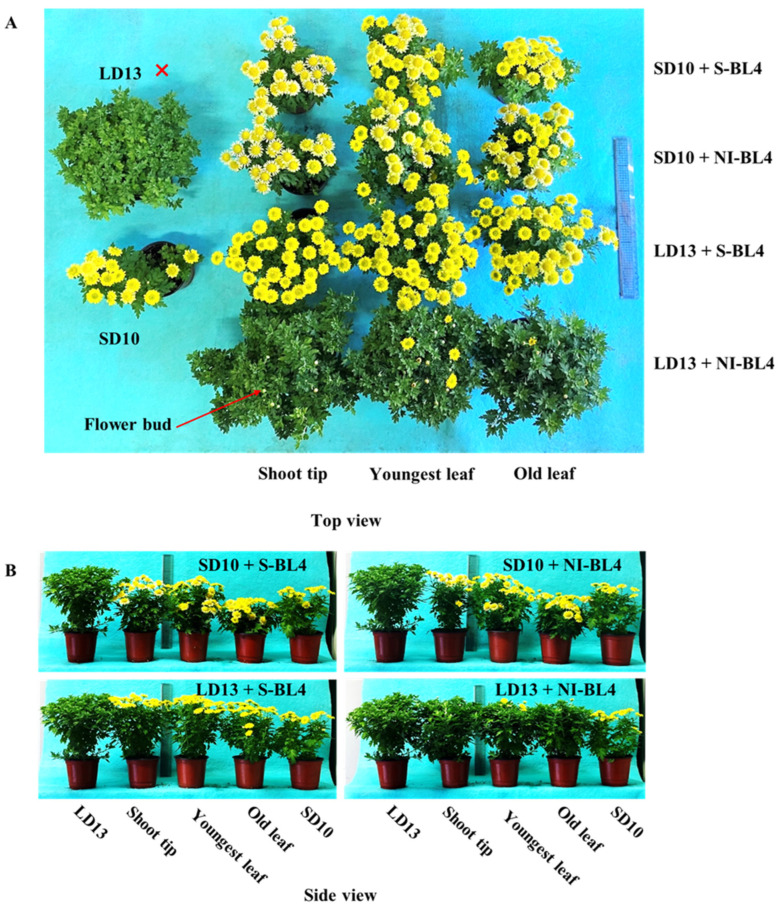
Morphology and flowering of ‘Gaya Glory’ grown under different lighting positions of supplemental or night-interruptional blue light for 60 days. Top (**A**) and side (**B**) views. The “❌” means non-flowered treatment.

**Figure 2:**
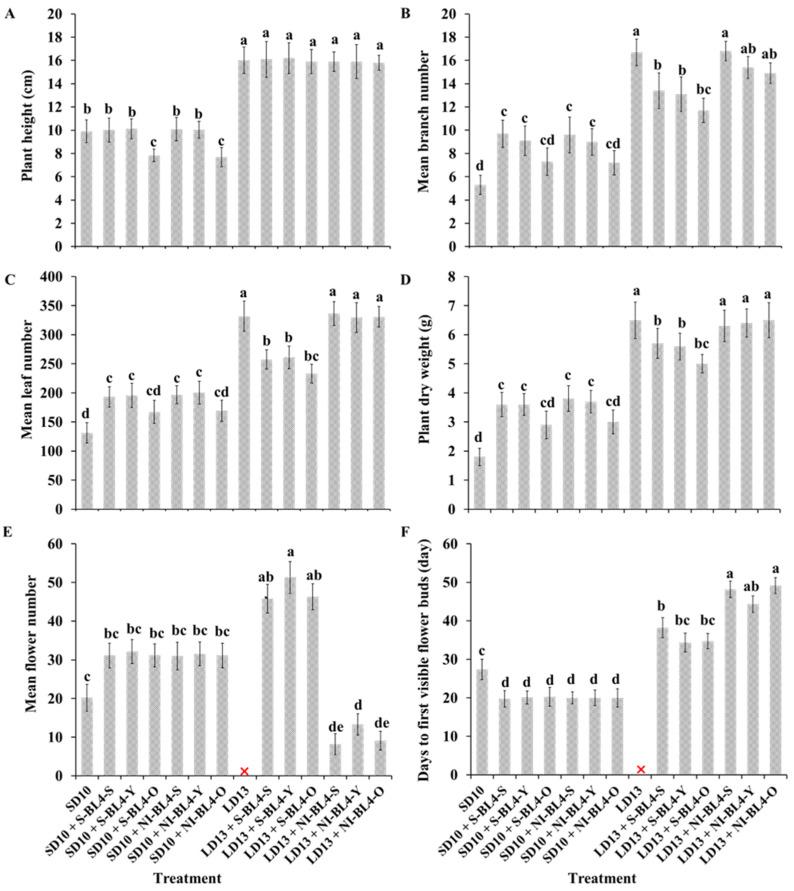
Measurements of morphological and growth parameters (**A**–**F**) of ‘Gaya Glory’ grown under different lighting positions of supplemental or night-interruptional blue light for 60 days. The “❌” means non-flowered treatment. Shoot tip, S; the youngest leaf, Y; and the old leaf, O. Different lowercase letters indicate significant differences within treatments by Duncan’s multiple range test at *p* ≤ 0.05. Vertical bars indicate the means ± standard error (n = 9).

The results indicate that the positioning of S-BL4 or NI-BL4, as well as the age of leaves (or tissues) exposed to the photoperiodic light treatment, significantly impacted the morphological characteristics of the chrysanthemum ‘Gaya Glory’ (Figure 1). In general, the LD13 conditions promoted greater vegetative growth, including increased plant height and a higher number of branches and leaves per plant, compared to the SD10 periods. The positioning of S-BL4 or NI-BL4 had no effect on plant height during LD13 conditions, or in SD10 periods when S-BL4 or NI-BL4 were shed onto the shoot tip and youngest leaf. However, a significant decrease in plant height was observed in treatments of SD10 + S-BL4-O and SD10 + NI-BL4-O when compared with chrysanthemums grown under SD10 treatment (Figure 2A). Under SD10 conditions, the application of S-BL4 and NI-BL4 significantly enhanced the formation of branches and leaves in chrysanthemums, particularly when the blue light was directed to illuminate the shoot tip and the youngest leaf (Figure 2B,C). Comparison of LD13 + S-BL4-O revealed a slight increase in the number of branches and leaves in chrysanthemums with treatments of LD13 + S-BL4-S and LD13 + S-BL4-Y, although always less than those in the LD13 treatment (Figure 2B,C). Additionally, the irradiation site of LD13 + NI-BL4 had a minimal effect on the number of branches and leaves of chrysanthemums. Furthermore, the fluctuation pattern of chrysanthemum dry weight under different treatments corresponded to the number of leaves (Figure 2D). Under LD13 conditions, the flowering-related traits were significantly impacted when the S-BL4 or NI-BL4 was shed onto the youngest leaf. However, these differences were not observed in the SD10 environments. At the harvest stage, with the exception of the non-flowered LD13 treatment, it was observed that the LD13 + S-BL4 irradiation on the youngest leaf induced the highest number of flowers, followed by irradiation onto the shoot tip and old leaf (Figure 2E). Additionally, LD13 + NI-BL4 irradiation, especially onto the shoot tip and old leaf, resulted in delayed flowering (Figure 2F). However, both SD10 + S-BL4 and SD10 + NI-BL4 treatments significantly accelerated flowering when compared to the SD10 treatment, regardless of whether they were applied to the shoot tip, youngest leaf, or old leaf.

### 2.2. Anatomical Structures of Leaves and Stems

**Figure 3:**
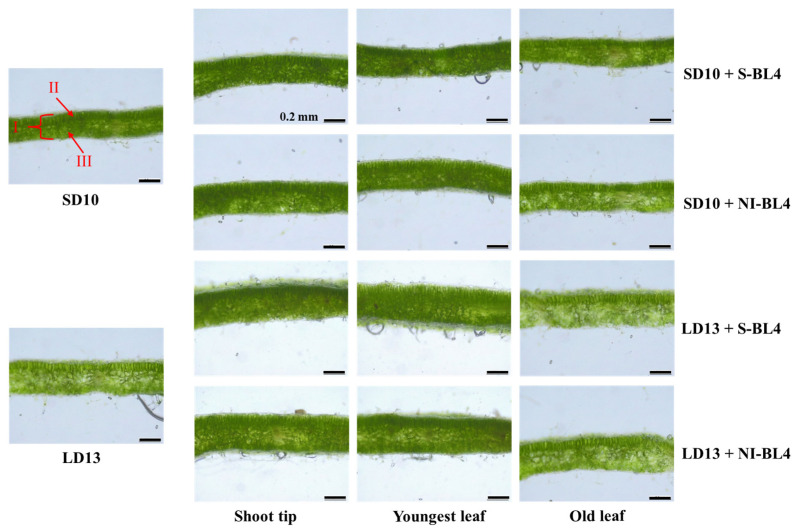
Leaf anatomy of ‘Gaya Glory’ grown under different lighting positions of supplemental or night-interruptional blue light for 60 days. I, leaf thickness; II, palisade tissue; III, spongy tissue. Bars indicate 0.2 mm.

**Figure 4:**
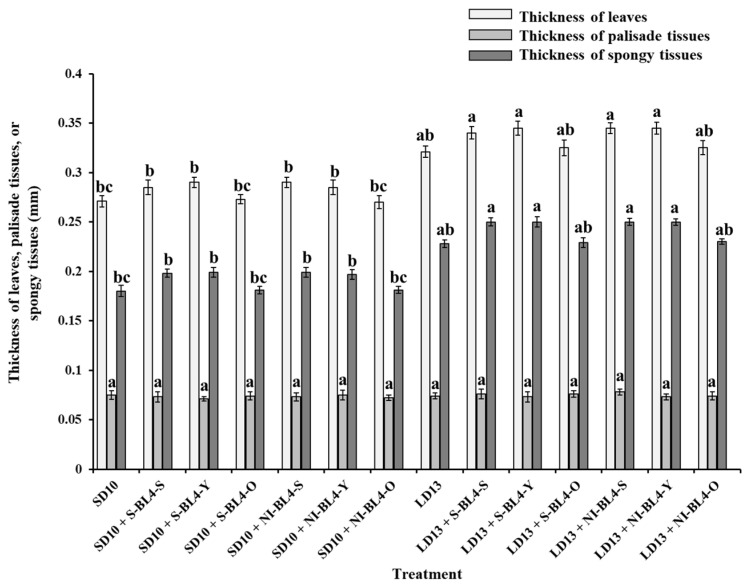
Measurements of leaf anatomy of ‘Gaya Glory’ grown under different lighting positions of supplemental or night-interruptional blue light for 60 days. Shoot tip, S; the youngest leaf, Y; and the old leaf, O. Different lowercase letters indicate significant differences within treatments by Duncan’s multiple range test at *p* ≤ 0.05. Vertical bars indicate the means ± standard error (n = 9).

The anatomical structures of leaves showed a strong response to the S-BL4 or NI-BL4 in different treatment positions. However, the LD13 conditions resulted in thicker leaves compared to the SD10 periods (Figure 3). Remarkably, chrysanthemum leaves exhibited well-developed palisade tissues characterized by tightly arranged layers of long cylindrical cells. Additionally, clearer and more compact structures of spongy tissues were observed when S-BL4 or NI-BL4 was applied to the shoot tip and youngest leaf, particularly under LD13 conditions. This phenomenon is expected to establish a solid foundation for leaf gas exchange and transpiration. Furthermore, the treatments of LD13 + S-BL4-S or -Y and LD13 + NI-BL4-S or -Y resulted in the maximum leaf thickness and spongy tissue thickness in chrysanthemum plants. However, there were subtle variations in the thickness of palisade tissues across all treatments (Figure 4).

**Figure 5:**
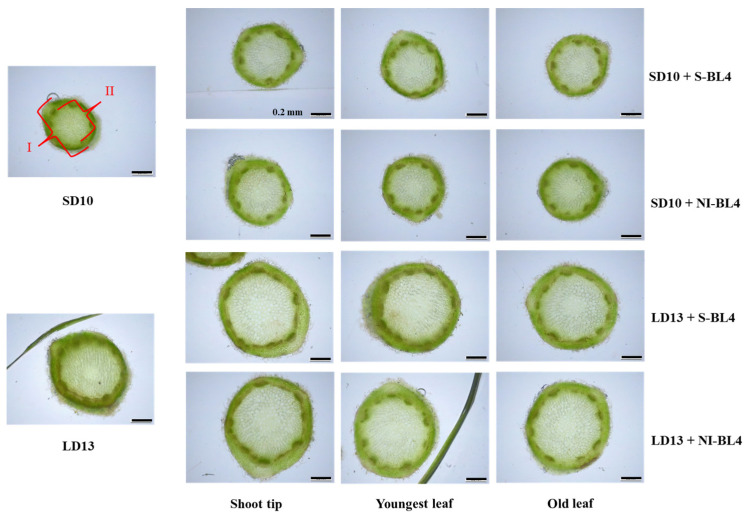
Stem anatomy of ‘Gaya Glory’ grown under different lighting positions of supplemental or night-interruptional blue light for 60 days. I, stem diameter; II, main pith diameter. Bars indicate 0.2 mm.

**Figure 6:**
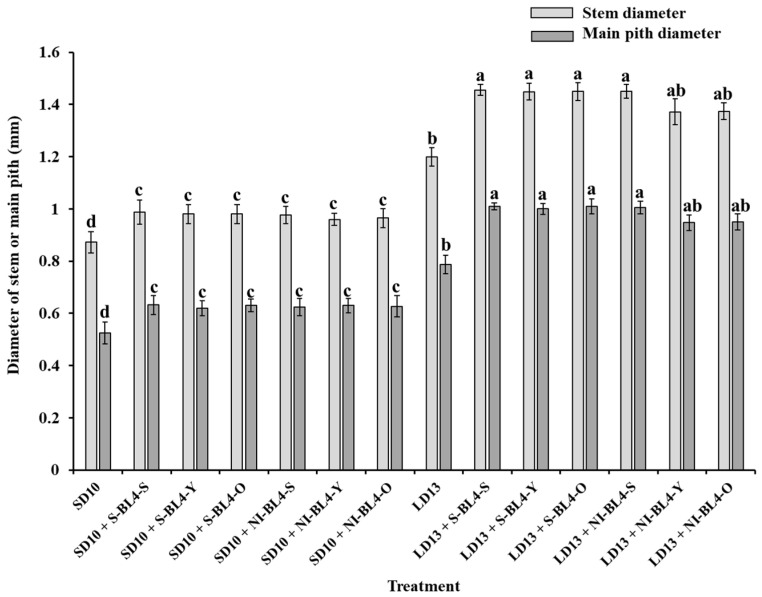
Measurements of stem anatomy of ‘Gaya Glory’ grown under different lighting positions of supplemental or night-interruptional blue light for 60 days. Shoot tip, S; the youngest leaf, Y; and the old leaf, O. Different lowercase letters indicate significant differences within treatments by Duncan’s multiple range test at *p* ≤ 0.05. Vertical bars indicate the means ± standard error (n = 9).

As illustrated in Figure 5, the use of positioned blue light significantly enhanced stem development in chrysanthemums, regardless of the photoperiod. Meanwhile, LD13 conditions were more favorable for stem development than SD10, irrespective of the lighting position of S-BL4 or NI-BL4 and leaf (or tissue) age. However, under SD10 conditions, there was no significant difference in both stem and main pith diameters observed between treatments with S-BL4 or NI-BL4 at the shoot tip, youngest leaf, or old leaf. Nonetheless, a slight decrease in the diameters of both the stem and main pith was noted in treatments of LD13 + NI-BL4-Y and LD13 + NI-BL4-O (Figure 6).

### 2.3. Gene Expression

**Figure 7:**
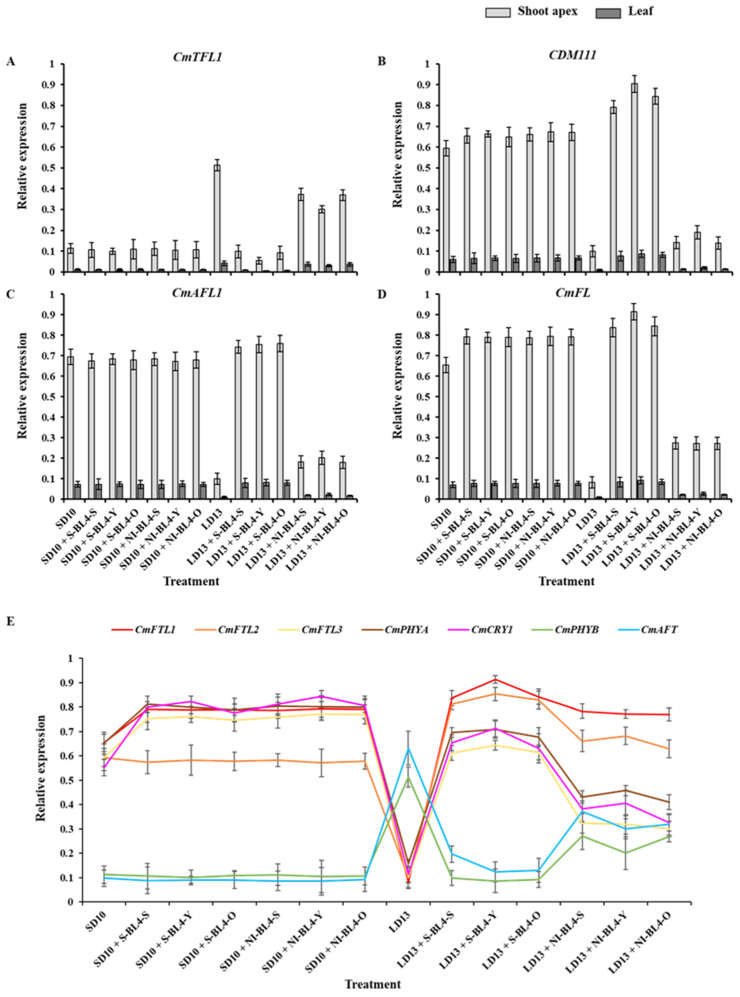
Expression patterns of flowering-related genes in chrysanthemum ‘Gaya Glory’ under different lighting positions of supplemental or night-interruptional blue light for 60 days. (**A**–**D**) The tissue-specific expression patterns of flowering-related genes in leaves and shoot apexes, and (**E**) the expression levels of flowering or photoreceptor-related genes in leaves. The top four mature leaves from the shoot apex and shoot apexes were harvested at 12:00 a.m. (4 h after lights-on) for RNA extraction and RT-PCR. Data were averagely normalized against the expression of *CmACTIN* and *CmEF1α*. The maximum value in each experiment was set to “1”. Shoot tip, S; the youngest leaf, Y; and the old leaf, O. Vertical bars indicate the means ± standard error of 9 biological replicates (n = 9).

In order to investigate the tissue-specific expression patterns of flowering-related genes in ‘Gaya Glory’ in response to the position of S-BL4 or NI-BL4, which are the chrysanthemum homologs of *Arabidopsis*, we selected and analyzed the anti-florigenic *TFL1/CEN*-like gene (*CmTFL1*) [37], as well as three well-characterized floral meristem identity genes *APETALA1* (*CDM111*), *FRUITFULL* (*CmAFL1*), and *LEAFY* (*CmFL*) [38,39] by qRT-PCR in leaves and shoot apexes, respectively (Figure 7A–D). After 60 days of exposure to the different treatments of S-BL4 or NI-BL4, the genes related to flower formation were found to be highly expressed in the shoot apices. However, their expression in leaves was significantly lower or barely detectable. The anti-florigenic gene *CmTFL1* showed higher expression in non-flowered or flowering-inhibited treatments LD13 and LD13 + NI-BL4-S, -Y, or -O, exhibiting an inverse relationship with flowering capacity (Figure 7A). Conversely, the three floral meristem identity genes *CDM111*, *CmAFL1*, and *CmFL* exhibited higher expression in flowering-promoted treatments SD10 + S-BL4/NI-BL4-S, -Y, or -O and LD13 + S-BL4-S, -Y, or -O while showing significantly lower expression in non-flowered or flowering-inhibited treatments of LD13 and LD13 + NI-BL4-S, -Y, or -O (Figure 7B–D).

The expression pattern of photoreceptor or flowering-related homologs of *Arabidopsis* in ‘Gaya Glory’ leaves after 60 days of positioned blue light treatments was also investigated (Figure 7E). The *FT*-like genes (*CmFTL1*, *CmFTL2*, and *CmFTL3*) [30,40], anti-florigenic FT/TFL1 family *TFL1/CEN/BFT*-like gene (*CmAFT*) [41], and three photo-receptor genes [*Phytochrome A* (*CmPHYA*), *Phytochrome B* (*CmPHYB*), and *Cryptochrome 1* (*CmCRY1*)] [29,30] were selected. The expression patterns of these genes can be broadly categorized into three types: (1) Specifically, the florigen gene *CmFTL3* and two photoreceptor genes—*CmPHYA* and *CmCRY1*—in ‘Gaya Glory’ leaves showed high expression levels in treatments of SD10 + S-BL4 and SD10 + NI-BL4, as well as in LD13 + S-BL4, particularly when the positioned blue light was directed onto the youngest leaf. In contrast, their expression levels were generally lower in LD13 + NI-BL4 and barely detectable in non-flowered LD13 treatment. (2) The expression levels of the anti-florigenic gene *CmAFT* and the photoreceptor gene *CmPHYB* were generally higher under delayed-flowering conditions (LD13 + NI-BL4) and extremely high in non-flowered LD13 treatment. (3) The expression levels of *CmFTL1* and *CmFTL2* were significantly higher under flowering-LD13-inductive conditions (LD13 + S-BL4 and LD13 + NI-BL4), but relatively lower in all SD10 treatments and markedly reduced in the non-flowered LD13 treatment. Moreover, it was observed that the youngest leaf exhibited greater sensitivity to the localized blue light. The constitutive expression of *CmFTL1* and *CmFTL2* in *Chrysanthemum morifolium* leaves indicates weak florigenic activity. It is suggested that two genes may function as an LD florigen similar to *RICE FLOWERING LOCUS T1* (*RFT1*) in rice, which is a facultative SDP [42].

## 3. Discussion

### 3.1. Plant Vegetative Growth

Light capture is heavily reliant on the light absorption characteristics of leaves. The pigment composition, thickness, tissue porosity, chloroplast orientation at different positions (ages) on the stem, and optical properties of leaves all contribute to the light absorption characteristics [43]. Light has an impact on the growth and development of plants through photoperiod, intensity, and quality [1]. Within the appropriate range of lighting intensity, plants tend to grow stronger and produce a higher yield when they receive a higher daily light integral (DLI). Therefore, for optimal vegetative growth, it is generally advisable to provide optimal light intensities over a longer photoperiod [44]. During periods of SD conditions, the duration of non-produced nighttime exceeds that of light, leading to a reduction in plant growth as they conserve accumulated sugars [45]. Compared with SD10 periods, the plants grown in LD13 conditions were incrementally taller (Figure 2A). A greater dry weight and thicker stem and leaves were also observed in LD13 conditions (Figure 2D, Figure 4 and Figure 6). The photoperiod is not only involved in energy provision but also in the regulation of branch or leaf formation. SDPs only grow nutritionally and do not flower in LD conditions [46]. Consistent with our results, the LDs caused more branches and leaves in the SDP chrysanthemum (Figure 2B,C).

The utilization of blue light during the photoperiod to restrain plant elongation has been previously documented in chrysanthemums [47,48,49]. Additionally, Khat Tak and Pearson reported that a low level of blue light during the photoperiod suppressed plant growth [50]. Our finding was that a significantly decreased plant height was observed in treatments of SD10 + S-BL4-O and SD10 + NI-BL4-O when compared with chrysanthemums grown under SD10 treatment (Figure 2A). This indicates that the application of S-BL4 or NI-BL4 on older leaves inhibits stem elongation, implying that the preferred sites for S-BL4 or NI-BL4 application may vary depending on the type of crops produced, such as cut flowers and potted flowers. These changes in stem elongation were attributed to a reduced number of leaves and branches, or a shortened time from treatment initiation to visible flower buds in all cases. Tewolde et al. reported that the length of tomato plant nodes decreased when red and blue LEDs were used in combination for inter-lighting on the lower canopy [51]. Inter-lighting, which involves directing light to the lower parts of plants, may be a practical method to effectively reduce plant height and control flowering in the lower plant canopy. The use of blue light inter-lighting may prove to be more effective than the combined red and blue light inter-lighting in controlling plant height. Additionally, it is suggested that S-BL4-O or NI-BL4-O could serve as a viable and environmentally friendly alternative to widely used plant growth retardants in the production of potted flowering plants. However, the lighting position of S-BL4 or NI-BL4 had no effect on plant height during LD13 conditions or in SD10 periods when S-BL4 or NI-BL4 shed onto the shoot tip and youngest leaf (Figure 2A), which might be because the blue light accounting for the proportion of the main white light is too weak.

Schuerger et al. observed that blue light has been shown to inhibit growth, alter stem anatomy, and induce morphological changes in the leaves of pepper plants [52]. Under SD10 conditions, the application of S-BL4 and NI-BL4 significantly promoted the number of branches and leaves in chrysanthemums, especially when the blue light was positioned to illuminate the shoot tip and the youngest leaf (Figure 2B,C). The maximum leaf thickness and spongy tissue thickness of chrysanthemum plants were noted under treatments of LD13 + S-BL4-S or -Y and LD13 + NI-BL4-S or -Y (Figure 4). While LD13 conditions were more conducive to stem development than SD10, no matter the lighting position of S-BL4 or NI-BL4 and the age of leaves (or tissues), only a slight decrease in the diameters of the stem and main pith was observed in treatments with LD13 + NI-BL4-Y and LD13 + NI-BL4-O (Figure 6). This suggests that the shoot tip was more sensitive to this additional weak blue light, and when NI-BL4 was illuminated on the shoot tip, it had a stronger effect on the development of plant stems. With the comparison of LD13 + S-BL4-O, a slight increase in the number of branches and leaves in chrysanthemums was observed in treatments with LD13 + S-BL4-S and LD13 + S-BL4-Y, but always less than those in the LD13 treatment (Figure 2B,C). A similar fluctuating pattern in the number of leaves and branches was observed across different treatments: an increase in flowers accompanied by a decrease in branches or leaves, indicating a competitive relationship between flower induction and leaf or branch formation. This was due to the rapid switch from vegetative growth to reproductive growth in all cases, except when S-BL4 or NI-BL4 shed onto the youngest leaf and shoo tip. The sensitivity of old leaves to NI appeared negligible when compared to that of the youngest leaf, particularly the shoot tip, aligning with established theory which suggests that florigen, a signal produced by the leaves, induces floral initiation at the shoot tip [10].

### 3.2. Photoperiodic Flowering and Gene Expression

According to Gao et al., the *CmTFL1* gene has been shown to enhance secondary branching in *Arabidopsis* and axillary bud development in *Chrysanthemum*, suggesting that high expression of *CmTFL1* promotes the development of lateral meristems in the stem [53]. Similar effects have been observed in other plant species with homologous *TFL1* genes. In *Lolium perenne* L., for example, the *LpTFL1* gene not only recovered the phenotype of *tfl1* mutants but also resulted in increased secondary branching and improved vegetative development [54]. Furthermore, the *AtTFL1* in *Arabidopsis*, the *PsTFL1* in *Prunus serotine*, and the *LjCEN1* gene in *Lotus japonicas* all demonstrated an increase in the number of branches and leaves [55,56,57]. This indicates that the *TFL1* gene plays a conserved role in regulating branching and leafing. Additionally, constitutive expression of *CsTFL1* significantly delayed flower formation under SD conditions in *Chrysanthemum seticuspe*. The function of *CmTFL1* was further confirmed with five transgenic lines, showing its impact on flower development in *Chrysanthemum morifolium* [37,58]. Similarly, transgenic *JcTFL1b*-RNAi Jatropha consistently exhibited earlier flowering [59]. In the current study, we observed a higher number of branches or leaves and a lower number of flowers (Figure 2B,C,E). The expression of *CmTFL1* was generally elevated under LD conditions, with particularly high levels in treatments showing a significant increase in branches and leaves, especially in LD13 and LD13 + NI-BL4 treatments (Figure 7A). Overall, the *CmTFL1* gene positively regulates branching and leafing while inhibiting flowering, resulting in a bushier plant.

It is widely accepted that inductive photoperiods stimulate the synthesis of a floral stimulus (“florigen”) in leaves. However, it has been theorized that an anti-florigenic signal produced in leaves may regulate photoperiodic floral induction; thus, an appropriate day length would lead to the suppression of an anti-florigen [24,60]. AFT, an anti-florigenic FT/TFL1 family protein, was identified in *Chrysanthemum seticuspe*. It has been convincingly demonstrated that the CsAFT protein functions as a systemic floral inhibitor—a signal generated in leaves under non-inductive conditions to inhibit flowering. Furthermore, the investigation of photoperiodic responses of *CsAFT*-RNAi plants supports the necessity of the anti-florigenic signal (CsAFT) for maintaining the vegetative state [37]. Therefore, the active flowering of chrysanthemum is primarily regulated by the photoperiodic control of florigen synthesis. Additionally, the *CmPHYB*-mediated anti-florigen *CmAFT* gene plays a predominant role in the obligatory photoperiodic flowering response in chrysanthemums, allowing for strict vegetative maintenance under non-inductive photoperiods (Figure 1, Figure 2 and Figure 7E). Chrysanthemum is an obligate SD plant that remains in a vegetative state in the absence of inductive LD conditions, such as LD13 treatment as observed in our study (Figure 1 and Figure 2). However, rice (*Oryza sativa*), a facultative SDP, may still flower even under non-inductive LD conditions. Two florigen genes (*Hd3a* and *RFT1*) in rice are activated based on the duration of daylight, with the RFT1 protein being identified as the LD florigen [42]. In chrysanthemums, *CmFTL1* may function similarly to *RFT1* in rice and serve as an LD florigen gene. It is reasonable to assume that the presence of residual *CmFTL3* and increased *CmFTL1* under unfavorable photoperiod conditions (Figure 7) ultimately leads to flowering to varying degrees in LD13 + S-BL4 and LD13 + NI-BL4, regardless of the light position (Figure 1A and Figure 2E). Therefore, it can be concluded that the photoperiodic flowering in chrysanthemums is co-regulated by both florigen and anti-florigen. The balanced synthesis of both factors determines the flowering response to photoperiodic light treatments.

Investigations into the expression of flowering-related genes in shoot apexes and leaves have significantly advanced our understanding of how to effectively control chrysanthemum flowering. Currently, three chrysanthemum orthologues of *FT* in *Chrysanthemum seticuspe*, namely *CsFTL1*, *CsFTL2*, and *CsFTL3*, have been identified. It was observed that the expression of *CsFTL3* plays a key role as a regulator in chrysanthemum photoperiodic flowering [61]. In the flower-inductive SD conditions, *CsFTFL3* was found to up-regulate floral-identity genes, thereby promoting the occurrence of flowering events in the shoot apical meristem (SAM) [61]. Furthermore, overexpression of *CsFTL3* led to the induction of chrysanthemum flowering under LD environments, suggesting that *CsFTL3* has the potential to induce chrysanthemum flowering even in photoperiod-unfavorable conditions. In the current study, following 60 days of photoperiodic treatments, it was observed that *CmFTL3*, along with two photoreceptor genes—*CmPHYA* and *CmCRY1* in *Chrysanthemum morifolium*, exhibited generally high levels of expression in SD10 + S-BL4 and SD10 + NI-BL4 treatments. Furthermore, their expression was notably enhanced when the lighting position was directed at the youngest leaf. In contrast, regardless of the lighting position, their expression levels were generally lower in LD13 + NI-BL4 treatments and barely detectable in non-flowered LD13 treatments (Figure 7). All of our findings suggest that the photoperiodic flowering of chrysanthemums is associated with photoreceptor-mediated control. Notably, our experiment demonstrates that plants with S-BL4 or NI-BL4 shed onto the youngest mature leaf and produced more flowers per plant compared to those in non-blue light treatments. This aligns with the concept of overflow metabolism, where the carbon demands linked to plant growth exceed carbohydrate production [62,63], resulting in a higher number of flowers per plant due to a relatively longer duration of vegetative growth.

## 4. Materials and Methods

### 4.1. Plant Materials and Growth Conditions

The chrysanthemum seedlings (*Chrysanthemum morifolium* Ramat. ‘Gaya Glory’, a qualitative SDP), with 8 ± 2 leaves per plant, were obtained from the “Flowers Breeding Research Institute, Gyeongnam Agricultural Research & Extension Services, Changwon, Gyeongnam, Korea” for this study. Each seedling was transplanted individually into a 10 × 10 cm plastic pot filled with commercial medium (BVB Medium, Bas Van Buuren Substrates, EN-12580, De Lier, the Netherlands). Following transplantation, the plants were transferred to an enclosed plant factory (770.0 cm long × 250.0 cm wide × 269.5 cm high, Green Industry Co. Ltd., Changwon, Korea) where the air temperature was maintained at a setpoint of 20 ± 2 °C and the relative humidity at 70 ± 10%. The plants were subjected to a 16 h photoperiod with a photosynthetic photon flux density (PPFD) of 270 ± 5 μmol·m^−2^·s^−1^ PPFD provided by fluorescent lamps (F48T12-CW-VHO, Philips Co., Ltd., Eindhoven, The Netherlands) under this LD conditions. An online CO_2_ sensor (Model No. GMT220 Carbocap, Vaisala, Vantaa, Finland) monitored the CO_2_ concentration of 1100-1200 ppm from a compressed gas tank to support plant photosynthesis. Air circulated horizontally through numerous evenly distributed holes in the cultivation rooms. After two weeks of acclimatization, the plants (with 12 ± 2 leaves per plant) were subjected to photoperiodic light treatments. Daily irrigation with the multipurpose nutrient solution [64] was from 8:30 to ~ 9:30 a.m. A randomized complete block design with 3 replications with 6 plants each was employed. The treatment locations in a controlled environment were randomly mixed between replications to minimize position effects.

### 4.2. Light Treatments

**Figure 8:**
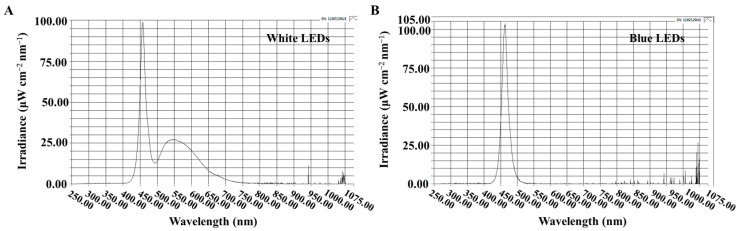
Spectral distribution of experimental light treatments: the daily white LEDs (range about 400~720 nm, and peaked at 452 nm) (**A**), and supplemental or night-interruptional blue LEDs (peaked at 450 nm) (**B**).

The critical day length required for flowering in ‘Gaya Glory’ used in this study is 12 h. Therefore, a 14 h or 11 h period of uninterrupted darkness (SD10 or LD13 treatment) was sufficient to initiate or inhibit flowering, respectively. The light duration started every day at 8:00 a.m. A light intensity of 300 ± 5 μmol·m^−2^·s^−1^ PPFD via white LEDs (MEF50120, More Electronics Co. Ltd., Changwon, Korea) (Figure 8A) was supplied during the photoperiod to grow the plants.

**Figure 9:**
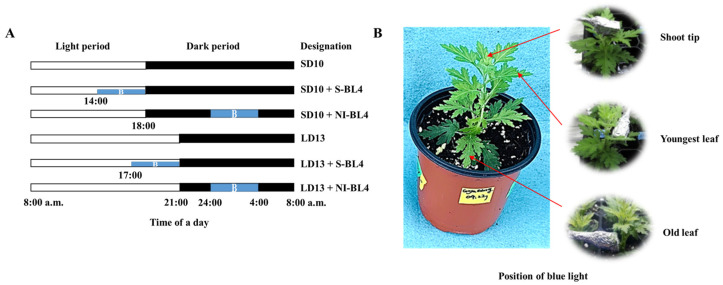
Experimental light schemes employed in this study (**A**). The treatment position of supplemental or night-interruptional blue light (**B**).

According to our previous study, the photoperiodic flowering and physiology of chrysanthemums are sensitive to the 30 μmol·m^−2^·s^−1^ PPFD of S-BL [36]. Thus, the blue LEDs with 30 μmol·m^−2^·s^−1^ PPFD intensity (Figure 8B) were used for 4 h either (1) to supplement the white LEDs at the end of the SD10 (SD10 + S-BL4) and LD13 (LD13 + S-BL4), or (2) to provide night interruption in the SD10 (SD10 + NI-BL4) and LD13 (LD13 + NI-BL4) (Figure 9A). The SD10 and LD13 served as controls. The S-BL4 or NI-BL4 was positioned to illuminate either the shoot tip (S), the youngest leaf (Y) (vigorously growing the third leaf from the shoot tip), or the old leaf (O) (third leaf from the stem base) (Figure 9B) [34]. In the text, they will be denoted as follows: SD10 + S-BL4-S, -Y, or -O; SD10 + NI-BL4-S, -Y, or -O; LD13 + S-BL4-S, -Y, or -O; and LD13 + NI-BL4-S, -Y, or -O. To minimize interference from other light sources and to illuminate specific areas with the S-BL4 or NI-BL4, a chip of specified LED light was securely fixed inside a rectangular column (20 mm × 20 mm × 40 mm in length) made of black cardboard wrapped with reflective aluminum foil (Figure 9B). The average light intensity of each treatment was measured using a quantum radiation probe (FLA 623 PS, ALMEMO, Holzkirchen, Germany) at a distance of 20 cm above the bench top and was adjusted to be consistent before the initiation of S-BL4 or NI-BL4 treatments. The light spectral distribution was recorded at 1 nm wavelength intervals using a hand-held spectroradiometer (MK550T, UPRtek, Miaoli, Taiwan, China; wavelength detection ranges from 200 to 1000 nm) positioned 25 cm above the bench top.

### 4.3. Measurements of Growth Parameters

The plant growth parameters, including plant height, number of branches, leaves, and flowers per plant, as well as plant dry weight, were recorded after 60 days of light treatments. The days to visible flower buds in each treatment were determined by counting the number of days from initiating the light treatment to the date when the first flower bud appeared. The count of flowers per plant included both blooming flowers and visible flower buds at the harvest stage. Leaves with a length > 1 cm were counted to determine the total number of leaves per plant. The shoots were dried for 7 days at 65 °C in a Venticell-222 dry oven (MMM Medcenter Einrichtungen GmbH., Munich, Germany) before measuring their dry weights. Additionally, harvested samples were immediately placed in liquid nitrogen and then stored in a −80 °C refrigerator for further investigations.

### 4.4. Microscopic Observation of Leaf and Stem Anatomy

The chrysanthemum leaves and stems were harvested when relatively tender to minimize damage from chemical solutions. Leaf and stem samples were cut to appropriate thickness by freehand slicing for fixation and observed directly without staining. The top four mature leaves and 3 cm length of the main stem from the middle part were collected from separate plants, with 9 bio-replicates per treatment. The leaf and stem segments were excised and fixed at 4 °C for 1~2 days in formalin-acetic acid-alcohol (FAA) solution, dehydrated in a series of ethanol solutions, and then examined with bright field microscopy (IX81, Olympus, Shinjuku, Tokyo, Japan). Cross-sectional images were taken and measured for thickness using ImageJ (ImageJ 1.48v, NIH, Bethesda, MD, USA). The specific processing method is detailed in [36].

### 4.5. Verification by Real-Time Quantitative PCR

Total RNA was extracted using the RNeasy Plant Mini Kit (Takara Bio Inc., Tokyo, Japan) and treated with RNase-free DNase (Takara Bio Inc., Tokyo, Japan) following the manufacturer’s instructions. A PrimeScript ^®^ Reverse Transcriptase (Takara Bio Inc., Tokyo, Japan) was utilized to synthesize cDNA from 1 μg of total RNA according to the manufacturer’s protocol. The cDNA was diluted 10-fold, and 5 μL was used in 15 μL quantitative RT-PCR (qRT-PCR) reactions with SYBR Premix Ex Taq™ II (Takara Bio Inc., Tokyo, Japan), conducted in a Roche Light Cycler 96 real-time fluorescence quantitative PCR instrument (Roche, Basel, Switzerland). The relative expression levels of each gene were determined using the 2^−∆∆Ct^ method [65]. In our study, the chrysanthemum homologs of Arabidopsis were denoted as “*Cm + gene*”. Data were normalized against the expression of reference genes *CmACTIN* and *CmEF1α* (*elongation factor 1α*) [30,66]. The primer sequences and PCR conditions used in the analyses can be found in Table 1. Each experiment included 3 technical replicates and 3 biological replicates.

### 4.6. Statistical Analysis

In this study, all plants were randomly sampled, and the data were processed, plotted, and statistically analyzed using Excel 2016 and the DPS software package (DPS for Windows, 2009). Significant differences among the treatments were assessed by ANOVA, followed by Duncan’s multiple range test at a probability (*p*) ≤ 0.05 with the SAS statistical program (Statistical Analysis System, V. 9.1, Cary, NC, USA). The differences between each treatment were tested by Student’s *t*-test (*p*) ≤ 0.05. Additionally, the experimental assays used to obtain all results were repeated 9 times and presented as the mean ± standard error.

## 5. Conclusions

The results indicated that the lighting position of S-BL4 or NI-BL4 and the age of leaves (or tissues) exposed to the photoperiodic light treatment notably affected the vegetative growth and photoperiodic flowering in chrysanthemums. According to the current study: (1) In general, vegetative growth was more sensitive to photoperiod rather than lighting position, while, during the same photoperiod, the promotion in it was stronger when the light position of S-BL4 or NI-BL4 was shoot tip or the youngest leaf. (2) The photoperiodic flowering of SDPs—chrysanthemums—comprehensively responded to the photoperiod combined with blue light positioning. Peculiarly, when they were exposed to the flowering-inhibited LD13 environments, the S-BL4 or NI-BL4 shed onto the leaves, especially on the youngest leaves, which significantly affected flowering. Based on these findings, we hope that the application of positioned S-BL or NI-BL could serve as a practical method for controlling plant appearance and photoperiodic flowering in the production of floricultural crops.

## Figures and Tables

**Table 1:** PCR conditions and primers used to quantify gene expression.

Name	Accession Number	Forward Primer (5′ to 3′)	Reverse Primer (5′ to 3′)
*CmACTIN*	AB205087	GATGACGCAGATCATGTTCG	AGCATGTGGAAGTGCATACC
*CmEF1α*	AB548817	CTTGTTGCTTGATGACTGTGG	CTTGTTGCTTGATGACTGTGG
*CmTFL1*	AB839767	CCATCATCAAGGCACAATTTCA	TTTCCCTTTGGCAGTTGAAGAA
*CDM111*	AY173054	GGTCTCAAGAATATTCGCAC	TCATTAGTCATCCCATCAGC
*CmAFL1*	AB451218	CAAGCTCAACCATCAATAGTC	TGCAGCACATGAACGAGTAG
*CmFL*	AB451217	CATTGATGCCATATTTAACTC	ACACGGATCATTCATTGTATA
*CmFTL1*	AB679270	AATCGTGTGCTATGAGAGCC	GCTTGTAACGTCCTCTTCATGC
*CmFTL2*	AB679271	ATGTGTTATTCCGGCAATTGGGTCG	AAATATGCATTTGTAACGTCATGTG
*CmFTL3*	AB679272	GGGAAAGTGGATTTGGTGGACG	GTCTTACAATTTGGTACTGTCG
*CmAFT*	AB839766	CAAGCAAAAAGCAAGGCAATCA	CAACCGGTAACCCCAAGTCATT
*CmPHYA*	AB733629	TGGAAGCAGTATGGATGCAA	TCGCAGGTATTGCACATCTC
*CmPHYB*	AB733630	TCCAAGAGGGTCATTTGGAG	ACCTGGCTAACCACAGCATC
*CmCRY1*	NM-116961	CGTAAGGGATCACCGAGTAAAG	CTTTTAGGTGGGAGTTGTGGAG
PCR Conditions		PCR was performed with an initial denaturing step at 95 °C for 5 min, followed by 40 cycles at 95 °C for 5 s, 60 °C for 20 s, 72 °C for 30 s, and 72 °C for 10 min to final extension. Fluorescence was quantified after the incubation at 72 °C.	

## Data Availability

No new data were created or analyzed in this study. Data sharing is not applicable to this article.

## References

[1] Kami C., Lorrain S., Hornitschek P., Fankhauser C. (2010). Light-regulated plant growth and development. Curr. Top. Dev. Biol..

[2] Leopold A. (1951). Photoperiodism in plants. Q. Rev. Biol..

[3] Cockshull K.E. (2019). Chrysanthemum morifolium. Handbook of Flowering.

[4] Lin C. (2000). Photoreceptors and regulation of flowering time. Plant Physiol..

[5] Takemiya A., Inoue S.-I., Doi M., Kinoshita T., Shimazaki K.-i. (2005). Phototropins promote plant growth in response to blue light in low light environments. Plant Cell.

[6] Kimura M., Kagawa T. (2006). Phototropin and light-signaling in phototropism. Curr. Opin. Plant Biol..

[7] López-Juez E., Bowyer J.R., Sakai T. (2007). Distinct leaf developmental and gene expression responses to light quantity depend on blue-photoreceptor or plastid-derived signals, and can occur in the absence of phototropins. Planta.

[8] Chailakhyan M.K. (1936). About the mechanism of the photoperiodic response. Dokl Akad Nauk SSSR.

[9] Kobayashi Y., Weigel D. (2007). Move on up, it’s time for change—Mobile signals controlling photoperiod-dependent flowering. Gene. Dev..

[10] Zeevaart J.A. (2008). Leaf-produced floral signals. Curr. Opin. Plant Biol..

[11] Turnbull C. (2011). Long-distance regulation of flowering time. J. Exp. Bot..

[12] McGarry R.C., Ayre B.G. (2012). Manipulating plant architecture with members of the CETS gene family. Plant Sci..

[13] Abe M., Kobayashi Y., Yamamoto S., Daimon Y., Yamaguchi A., Ikeda Y., Ichinoki H., Notaguchi M., Goto K., Araki T. (2005). FD, a bZIP protein mediating signals from the floral pathway integrator FT at the shoot apex. Science.

[14] Wigge P.A., Kim M.C., Jaeger K.E., Busch W., Schmid M., Lohmann J.U., Weigel D. (2005). Integration of spatial and temporal information during floral induction in Arabidopsis. Science.

[15] Kobayashi Y., Kaya H., Goto K., Iwabuchi M., Araki T. (1999). A pair of related genes with antagonistic roles in mediating flowering signals. Science.

[16] Kardailsky I., Shukla V.K., Ahn J.H., Dagenais N., Christensen S.K., Nguyen J.T., Chory J., Harrison M.J., Weigel D. (1999). Activation tagging of the floral inducer FT. Science.

[17] Yamaguchi A., Kobayashi Y., Goto K., Abe M., Araki T. (2005). TWIN SISTER OF FT (TSF) acts as a floral pathway integrator redundantly with FT. Plant Cell Physiol..

[18] Bradley D., Ratcliffe O., Vincent C., Carpenter R., Coen E. (1997). Inflorescence commitment and architecture in Arabidopsis. Science.

[19] Mimida N., Goto K., Kobayashi Y., Araki T., Ahn J.H., Weigel D., Murata M., Motoyoshi F., Sakamoto W. (2001). Functional divergence of the TFL1-like gene family in Arabidopsis revealed by characterization of a novel homologue. Genes Cells.

[20] Yoo S.J., Chung K.S., Jung S.H., Yoo S.Y., Lee J.S., Ahn J.H. (2010). BROTHER OF FT AND TFL1 (BFT) has TFL1-like activity and functions redundantly with TFL1 in inflorescence meristem development in Arabidopsis. Plant J..

[21] Xi W., Liu C., Hou X., Yu H. (2010). MOTHER OF FT AND TFL1 regulates seed germination through a negative feedback loop modulating ABA signaling in Arabidopsis. Plant Cell.

[22] Conti L., Bradley D. (2007). TERMINAL FLOWER1 is a mobile signal controlling Arabidopsis architecture. Plant Cell.

[23] Huang N.C., Jane W.N., Chen J., Yu T.S. (2012). Arabidopsis thaliana CENTRORADIALIS homologue (ATC) acts systemically to inhibit floral initiation in Arabidopsis. Plant J..

[24] Thomas B., Vince-Prue D. (1996). Photoperiodism in Plants.

[25] Lang A., Chailakhyan M.K., Frolova I. (1977). Promotion and inhibition of flower formation in a dayneutral plant in grafts with a short-day plant and a long-day plant. Proc. Natl. Acad. Sci. USA.

[26] Tanaka T. (1967). Studies on the regulation of Chrysanthemum flowering with special reference to plant regulators I. The inhibiting action of non-induced leaves on floral stimulus. J. Jpn. Soc. Hortic. Sci..

[27] Zheng Q., Weng Q., Huang L., Wang K., Deng J., Jiang R., Ye Z., Gan M. (2018). A new source of multi-spectral high spatial resolution night-time light imagery—JL1-3B. Remote Sens. Environ..

[28] Yamada A., Tanigawa T., Suyama T., Matsuno T., Kunitake T. (2008). Night break treatment using different light sources promotes or delays growth and flowering of Eustoma grandiflorum (Raf.) Shinn. J. Jpn. Soc. Hortic. Sci..

[29] Park Y.G., Muneer S., Jeong B.R. (2015). Morphogenesis, flowering, and gene expression of Dendranthema grandiflorum in response to shift in light quality of night interruption. Int. J. Mol. Sci..

[30] Higuchi Y., Sumitomo K., Oda A., Shimizu H., Hisamatsu T. (2012). Day light quality affects the night-break response in the short-day plant chrysanthemum, suggesting differential phytochrome-mediated regulation of flowering. J. Plant Physiol..

[31] Park Y.G., Jeong B.R. (2020). How supplementary or night-interrupting low-intensity blue light affects the flower induction in chrysanthemum, a qualitative short-day plant. Plants.

[32] Park Y.G., Jeong B.R. (2019). Night interruption light quality changes morphogenesis, flowering, and gene expression in Dendranthema grandiflorum. Hortic. Environ. Biotechnol..

[33] SharathKumar M., Heuvelink E., Marcelis L.F., Van Ieperen W. (2021). Floral induction in the short-day plant chrysanthemum under blue and red extended long-days. Front. Plant Sci..

[34] Park Y.G., Jeong B.R. (2020). Both the quality and positioning of the night interruption light are important for flowering and plant extension growth. J. Plant Growth Regul..

[35] Yang J., Song J., Jeong B.R. (2022). Blue light supplemented at intervals in long-day conditions intervenes in photoperiodic flowering, photosynthesis, and antioxidant properties in chrysanthemums. Antioxidants.

[36] Yang J., Song J., Jeong B.R. (2022). The flowering of SDP chrysanthemum in response to intensity of supplemental or night-interruptional blue light is modulated by both photosynthetic carbon assimilation and photoreceptor-mediated regulation. Front. Plant Sci..

[37] Higuchi Y., Narumi T., Oda A., Nakano Y., Sumitomo K., Fukai S., Hisamatsu T. (2013). The gated induction system of a systemic floral inhibitor, antiflorigen, determines obligate short-day flowering in chrysanthemums. Proc. Natl. Acad. Sci. USA.

[38] Shchennikova A.V., Shulga O.A., Immink R., Skryabin K.G., Angenent G.C. (2004). Identification and characterization of four chrysanthemum MADS-box genes, belonging to the APETALA1/FRUITFULL and SEPALLATA3 subfamilies. Plant Physiol..

[39] Li T., Niki T., Nishijima T., Douzono M., Koshioka M., Hisamatsu T. (2009). Roles of *CmFL*, *CmAFL1*, and *CmSOC1* in the transition from vegetative to reproductive growth in *Chrysanthemum morifolium* Ramat. J. Hortic. Sci. Biotech..

[40] Sun J., Wang H., Ren L., Chen S., Chen F., Jiang J. (2017). CmFTL2 is involved in the photoperiod-and sucrose-mediated control of flowering time in chrysanthemum. Hortic. Res..

[41] Zhao K., Li S., Jia D., Xing X., Wang H., Song A., Jiang J., Chen S., Chen F., Ding L. (2022). Characterization of the MADS-Box Gene CmFL3 in chrysanthemum. Agronomy.

[42] Komiya R., Yokoi S., Shimamoto K. (2009). A gene network for long-day flowering activates RFT1 encoding a mobile flowering signal in rice. Development.

[43] Van Ieperen W. Plant morphological and developmental responses to light quality in a horticultural context. Proceedings of the VII International Symposium on Light in Horticultural Systems 956.

[44] Pearcy R.W. (1989). Radiation and light measurements. Plant Physiological Ecology: Field Methods and Instrumentation.

[45] Gent M.P. (2018). Dynamic carbohydrate supply and demand model of vegetative growth: Response to temperature, light, carbon dioxide, and day length. Agronomy.

[46] Garner W.W. (1933). Comparative responses of long-day and short-day plants to relative length of day and night. Plant Physiol..

[47] Oyaert E., Volckaert E., Debergh P. (1999). Growth of chrysanthemum under coloured plastic films with different light qualities and quantities. Sci. Hortic..

[48] Kim H.-H., Goins G.D., Wheeler R.M., Sager J.C. (2004). Green-light supplementation for enhanced lettuce growth under red-and blue-light-emitting diodes. HortScience.

[49] Shimizu H., Ma Z., Tazawa S., Douzono M., Runkle E., Heins R. Blue light inhibits stem elongation of chrysanthemum. Proceedings of the V International Symposium on Artificial Lighting in Horticulture 711.

[50] Khattak A.M., Pearson S. (2006). Spectral filters and temperature effects on the growth and development of chrysanthemums under low light integral. Plant Growth Regul..

[51] Tewolde F.T., Lu N., Shiina K., Maruo T., Takagaki M., Kozai T., Yamori W. (2016). Nighttime supplemental LED inter-lighting improves growth and yield of single-truss tomatoes by enhancing photosynthesis in both winter and summer. Front. Plant Sci..

[52] Schuerger A.C., Brown C.S., Stryjewski E.C. (1997). Anatomical features of pepper plants (*Capsicum annuum* L.) grown under red light-emitting diodes supplemented with blue or far-red light. Ann. Bot..

[53] Gao Y., Gao Y., Wu Z., Bu X., Fan M., Zhang Q. (2019). Characterization of TEMINAL FLOWER1 homologs CmTFL1c gene from *Chrysanthemum morifolium*. Plant Mol. Biol..

[54] Jensen C.S., Salchert K., Nielsen K.K. (2001). A TERMINAL FLOWER1-like gene from perennial ryegrass involved in floral transition and axillary meristem identity. Plant Physiol..

[55] Ratcliffe O.J., Amaya I., Vincent C.A., Rothstein S., Carpenter R., Coen E.S., Bradley D.J. (1998). A common mechanism controls the life cycle and architecture of plants. Development.

[56] Wang Y., Pijut P.M. (2013). Isolation and characterization of a TERMINAL FLOWER 1 homolog from Prunus serotina Ehrh. Tree Physiol..

[57] Guo X., Zhao Z., Chen J., Hu X., Luo D. (2006). A putative CENTRORADIALIS/TERMINAL FLOWER 1-like gene, Ljcen1, plays a role in phase transition in Lotus japonicus. J. Plant Physiol..

[58] Higuchi Y., Hisamatsu T. (2015). CsTFL1, a constitutive local repressor of flowering, modulates floral initiation by antagonising florigen complex activity in chrysanthemum. Plant Sci..

[59] Li C., Fu Q., Niu L., Luo L., Chen J., Xu Z.-F. (2017). Three TFL1 homologues regulate floral initiation in the biofuel plant Jatropha curcas. Sci. Rep..

[60] Lang A., Melchers G. (1943). Die photoperiodische Reaktion von Hyoscyamus niger. Planta.

[61] Oda A., Narumi T., Li T., Kando T., Higuchi Y., Sumitomo K., Fukai S., Hisamatsu T. (2012). CsFTL3, a chrysanthemum FLOWERING LOCUS T-like gene, is a key regulator of photoperiodic flowering in chrysanthemums. J. Exp. Bot..

[62] Matsuki M. (1996). Regulation of plant phenolic synthesis: From biochemistry to ecology and evolution. Aust. J. Bot..

[63] Park Y.G., Oh H.J., Jeong B.R. (2013). Growth and anthocyanin concentration of Perilla frutescens var. acuta Kudo as affected by light source and DIF under controlled environment. Hortic. Environ. Biotechnol..

[64] Yang J., Song J., Jeong B.R. (2022). Low-intensity blue light supplemented during photoperiod in controlled environment induces flowering and antioxidant production in kalanchoe. Antioxidants.

[65] Livak K.J., Schmittgen T.D. (2001). Analysis of relative gene expression data using real-time quantitative PCR and the 2^−ΔΔCT^ method. Methods.

[66] Gu C., Chen S., Liu Z., Shan H., Luo H., Guan Z., Chen F. (2011). Reference gene selection for quantitative real-time PCR in Chrysanthemum subjected to biotic and abiotic stress. Mol. Biotechnol..

